# Response to “Letter to the editor on: Tooth loss, diet quality, and cognitive decline: A 15-year longitudinal study”

**DOI:** 10.1016/j.jnha.2025.100667

**Published:** 2025-08-29

**Authors:** Lewis Winning, Gerard J. Linden

**Affiliations:** a Dublin Dental University Hospital, Trinity College Dublin, Lincoln Place, Dublin 2, D02 F859, Ireland; b Centre for Public Health, School of Medicine, Dentistry and Biomedical Sciences, Queen’s University Belfast, Institute of Clinical Sciences, Royal Victoria Hospital, Belfast, BT12 6BA, United Kingdom

Dear Editor,

We appreciate the interest and thoughtful reflections from Wu et al. regarding our recent publication in The Journal of Nutrition, Health and Aging [1]. We thank the correspondents for highlighting several important points, which we address below. Overall, we agree with the observations and offer further clarification on the scope and methodological choices of our study.

First, we agree that a life-course perspective is highly valuable for framing oral health as a structural marker of disadvantage. Oral diseases, including dental caries, periodontitis, and their sequela of tooth loss, disproportionately affect socially disadvantaged groups and serve as cumulative indicators of health inequalities across the life course. This aligns with broader research arguing that oral health trajectories mirror lifetime exposures and socioeconomic conditions [2]. We acknowledge that exploring the upstream social determinants of oral health, such as early-life disadvantage or lifelong socioeconomic status, is a meaningful avenue for further inquiry especially in the context of investigating risk factors for future cognitive decline. However, incorporating a full life-course analysis was beyond the scope of our current work, which focused on diet quality as a mediator of the tooth loss-cognition relationship. We agree with Wu et al. that future studies would benefit from integrating life-course measures to untangle how structural disadvantages drive both oral and cognitive health outcomes [3].

Second, Wu et al. suggest modelling tooth count as a continuous or multi-level categorical variable rather than a dichotomous indicator. In our study, we defined significant tooth loss as having <20 remaining natural teeth, consistent with the World Health Organization’s definition of an inadequate “functional dentition” [4]. We chose this binary cutoff for its clinical relevance and to facilitate interpretation, especially since our aim was to investigate diet quality as a potential mediator in the tooth loss-cognitive decline relationship. That said, we acknowledge the limitation of this approach, and as Wu et al. suggest, modelling tooth number on a continuous scale or using flexible forms (e.g., splines) may capture gradients more precisely. In this cohort, however, the low number of participants included in the 15-year follow-up analysis (n = 628) constrained power for more granular categorisations and flexible modelling. We endorse further studies with larger and more diverse samples to examine dose-response patterns using continuous or non-linear specifications.

Third, regarding denture use and oral function: we agree with Wu et al. that simply adjusting for denture use (yes/no) is a crude proxy for masticatory function. In our analysis, we included denture use as a covariate primarily to account for some degree of oral functional status, acknowledging that it is an imperfect measure. This approach was consistent with prior epidemiological studies that have used denture status as a basic indicator of whether an individual has replaced missing teeth [5]. We recognise, however, that denture use alone does not capture important factors such as how well the dentures fit, how often they are worn, or the actual chewing efficiency they confer. These factors can significantly influence diet and nutrition in edentulous or partially edentulous individuals. Future research should consider assessing denture fit and usage, masticatory performance, or chewing ability tests to more directly quantify oral function. Incorporating such detailed oral functional metrics could help elucidate how effectively dental prostheses mitigate the impacts of tooth loss on diet and systemic health outcomes.

To conclude, we are grateful to Wu et al. for their constructive comments and for extending the dialogue on our study’s implications. We share their view that tackling oral health disparities over the life course and improving oral functional measures are important next steps in this field. Our study contributes to understanding the oral health-cognition relationship by examining diet quality as a key mediating pathway, and we appreciate the correspondents' suggestions for expanding this research framework. We extend our appreciation to the Journal of Nutrition, Health and Aging for facilitating this valuable scientific exchange and the opportunity to respond.

## Declaration of competing interest

None.
